# Specification of photonic circuits using quantum hardware description language

**DOI:** 10.1098/rsta.2011.0526

**Published:** 2012-11-28

**Authors:** Nikolas Tezak, Armand Niederberger, Dmitri S. Pavlichin, Gopal Sarma, Hideo Mabuchi

**Affiliations:** 1 Edward L. Ginzton Laboratory, Stanford University, Stanford, CA 94305, USA; 2 SUPA, Department of Physics, University of Strathclyde, Glasgow G4 0NG, UK

**Keywords:** quantum control, photonics, quantum information, quantum optics, quantum feedback

## Abstract

Following the simple observation that the interconnection of a set of quantum optical input–output devices can be specified using structural mode VHSIC hardware description language, we demonstrate a computer-aided schematic capture workflow for modelling and simulating multi-component photonic circuits. We describe an algorithm for parsing circuit descriptions to derive quantum equations of motion, illustrate our approach using simple examples based on linear and cavity-nonlinear optical components, and demonstrate a computational approach to hierarchical model reduction.

## 1. Introduction

Ongoing advances in materials science, nanoscale synthesis and lithography are establishing key technical requirements for the fabrication of complex nanophotonic circuits. Critical applications for such circuitry can be foreseen in ultra-low-power photonic signal processing and interconnects, sensor networks and quantum information technology. Although current research in nanophotonics focuses largely on the physics of individual devices such as resonators and waveguides, there are substantial new challenges to be addressed in the analysis and design of circuits comprising large-scale networks of interconnected components with dynamic optical nonlinearities. For premier applications, quantum-optical network models will be required in order to capture fluctuations, coherence and entanglement effects that may be decisive drivers of the overall circuit performance. Suitable theoretical frameworks exist but require cumbersome algebraic manipulations for the derivation of multi-component models, pointing to the need for computer-aided paradigms for generating quantum-optical equations of motion from high-level circuit representations that can be manipulated more intuitively by circuit engineers. Simply put, we are rapidly approaching a new phase of research in quantum nonlinear photonics in which we will need user-friendly circuit design tools like those we have long exploited in classical electronics.

In this paper, we propose and demonstrate a modelling and simulation workflow based on schematic capture using a quantum hardware description language (QHDL) for nanophotonic circuits, which we will define as a proper subset of the standard VHSIC hardware description language (VHDL). Our approach uses a mixture of common open-source software packages and custom processing scripts to provide a high-level, modular interface to the quantum circuit ‘algebra’ of Gough & James [1,2] (which generalizes earlier work on cascaded open quantum systems by Carmichael [3] and by Gardiner [4]). The natural hierarchical organization of VHDL and the schematic capture workflow should facilitate future work on model reduction and design abstractions for nanophotonic circuits, which seems essential, given the extremely high dimension (variable count) associated with many-component quantum models.

In the following sections, we first review the formal setting of (**S**,**L**,*H*) component models and the concatenation and series and products as introduced by Gough & James, which have recently been used to derive quantum nonlinear photonic circuit models by hand or using custom-coded computer algebra scripts [5–8]. While we will restrict our attention here to linear and cavity nonlinear optics, it should be noted that the (**S**,**L**,*H*) formalism can in principle be used to describe hybrid circuits incorporating suitable spintronic, nanomechanical [9] and/or quantum-electronic components. Likewise, the approach we describe here could be extended straightforwardly to admit static Bogoliubov components as described in Gough [10]. We then review the proposed syntax of QHDL and illustrate its use in the specification of a simple interferometer as a network of elementary optical components. After describing methods that can be used to parse QHDL circuit descriptions to derive quantum equations of motion for analysis and/or numerical simulation, we illustrate the full schematic capture workflow using an example of constructing a bistable latch from cavity nonlinear optical components. The paper closes with a brief consideration of model reduction in the (**S**,**L**,*H*) context.

## 2. Modelling quantum circuitry

Within this section, we use {*Q*_*j*_,*j*=1,2,3,…,*N*} to denote individual quantum input–output components. We clearly distinguish between input and output ports and do not consider bi-directional ports, although for physical reasons every input port is assumed to have an associated output port and *vice versa*.

### (a) The circuit algebra

Our modelling workflow is based on the Gough–James synthesis results for open quantum systems [1,2], which provide a purely algebraic method to derive quantum Markov models for a network of interconnected quantum components.

A component with an equal number *n* of input and output channels is described by the parameter triplet (**S**,**L**,*H*), where *H* is the effective internal *Hamilton operator* for the system, **L**=(*L*_1_,*L*_2_,…,*L*_*n*_)^T^ the *coupling vector* and 
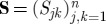
 the *scattering matrix*, whose elements are themselves operators.

Each element *L*_*k*_ of the coupling vector is given by an operator that describes the system's coupling to the *k*th input channel. Similarly, the elements *S*_*jk*_ of the scattering matrix are given by system operators describing the scattering between different field channels *j* and *k*. The only mathematical conditions on the parameters are that the Hamiltonian operator be self-adjoint and the scattering matrix be unitary:

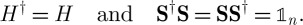


The master equation [11] corresponding to a given (**S**,**L**,*H*) model is
2.1
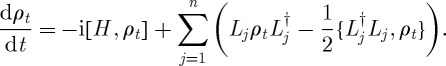
Here [*A*,*B*]≡*AB*−*BA* and {*A*,*B*}≡*AB*+*BA*, while *ρ*_*t*_ is a density matrix describing the evolving state of the internal degrees of freedom. It is also straightforward to obtain the quantum filtering equations [12,13] for stochastic simulation of a given (**S**,**L**,*H*) model.

While the scattering matrix elements *S*_*jk*_ do not appear in equation (2.1), they are required for the composition rules described later, which can be used to derive the overall parameter triplet for a network of interconnected quantum input–output components. The (**S**,**L**,*H*) circuit algebra plus simple correspondences such as equation (2.1) provide all that is needed to obtain overall equations of motion for complex photonic circuits.

Gough & James [2] have introduced two operations that allow for the construction of arbitrary networks of optical *feed-forward* circuits.

**Figure 1.**
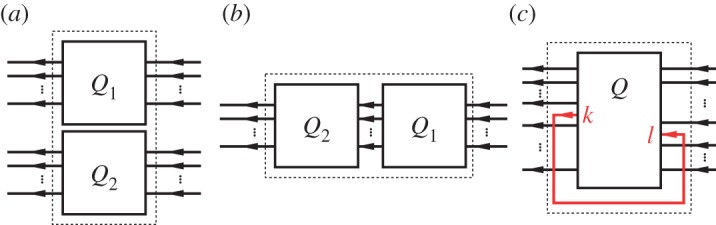
Basic operations of the Gough–James circuit algebra. (*a*) 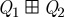; (*b*) 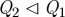; (*c*) [*Q*]_*k*→*l*_.

— The *concatenation* product (cf. figure 1*a*) describes a formal adjoining of two systems in which there is no optical scattering between the systems:
2.2
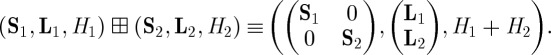
Note however that even without optical scattering, the two subsystems may interact via shared quantum degrees of freedom. A simple example of this scenario is given by a two-mode resonator (such as a ring resonator) with an atom that interacts with both optical modes, but in which there is no direct scattering between the modes.
— The *series* product (cf. figure 1*b*) describes a configuration in which two systems *Q*_*j*_=(**S**_*j*_,**L**_*j*_,*H*_*j*_), *j*=1,2 possessing an equal number of channels *n* are connected in such a manner that all output channels of *Q*_1_ are fed into the corresponding input channels of *Q*_2_. The resulting system is then given by
2.3
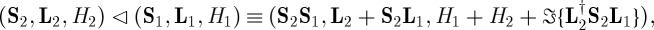
where we define the imaginary part of an operator as ℑ{*A*}≡(*A*−*A*^†^)/2i.

To make the network operations complete, one additional rule is required: the *feedback* operation (cf. figure 1*c*) describes the case where the *k*th output channel of a system with *n*≥2 channels is fed back into the *l*th input channel. The result is a system with *n*−1 channels:
2.4
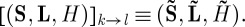
Formulae for the resulting parameter triplet are provided in appendix A.

Note that the series product can be expressed in terms of the concatenation and feedback operations (e.g. for two components with *n*=1, we have 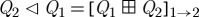), and consequently, the latter two operations are sufficient to perform all network calculations. However, the series product is a useful shorthand and allows for a more intuitive network expression.

For use in the following, we define the identity system with *n* channels
2.5
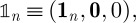
where 
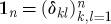
 is the identity matrix in *n* dimensions, as well as the channel permuting system
2.6
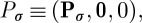
where the permutation matrix is defined by 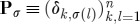. This definition ensures that 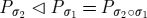.

### (b) The quantum hardware description language syntax

QHDL is a subset of structural VHDL [14], which we will use as a formal syntax for specifying photonic circuits in terms of interconnections among referenced quantum input–output components. These components can themselves represent composite networks of subcomponents, facilitating hierarchical approaches to photonic circuit design. It is useful to start with a set of basic components such as beamsplitters and phase-shifts, as well as linear and nonlinear cavity models with one or more coupling mirrors,^1^ which can be collected in a shared library file. The set of such *primitive* components within a QHDL software environment can of course be extended at any time.

Within the context of a single QHDL file, the exact physical model (parameter triplet) of any referenced component is left unspecified except for its external ports and parametric dependencies. This approach allows the circuit designer to operate at a high level of abstraction, facilitating last-minute substitution of alternative physical component models (including effective models with reduced simulation complexity) into a given interconnection topology.

**Figure 2.**
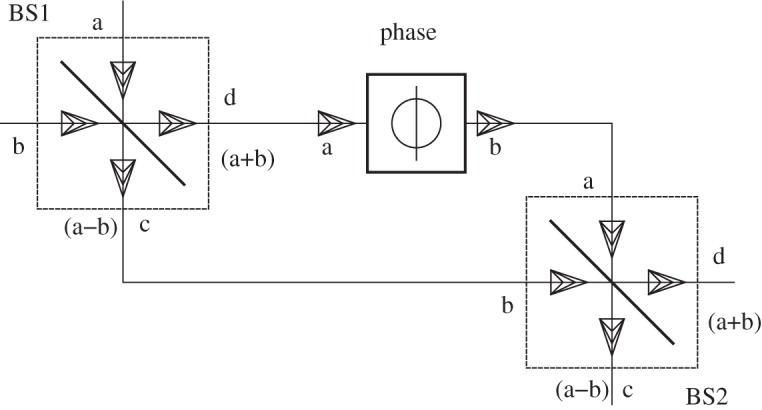
A basic Mach–Zehnder set-up.

In the following section, we will introduce the QHDL syntax by means of a very simple circuit that realizes a Mach–Zehnder interferometer (figure 2).

A QHDL file begins with the *entity* declaration, which defines the abstract interface of the circuit being specified: it specifies a list of named input and output ports (of the overall circuit), which are required in order for the circuit itself to be callable as a composite QHDL component, as well as any numeric parameters required for physical modelling. Note that we require that all input ports appear before all output ports.

**Listing 1.** Entity declaration.

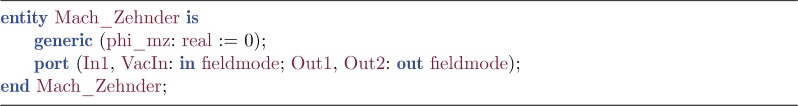

For this entity, we must then have one or more *architecture* declarations in the same QHDL file. These provide alternative ways of realizing the internal structure of the circuit. The architecture declaration consists of a head that specifies the interfaces of all *components* used in the architecture body and all internal *signals*. The component declarations are very similar to the entity declaration—they serve to define an interface for each subcomponent.

**Listing 2.** Architecture head.

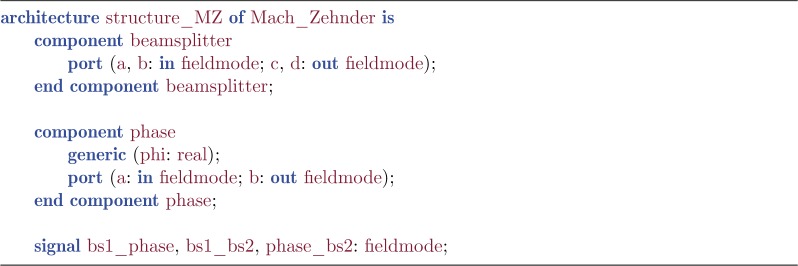

The architecture body then consists of a series of *instance* assignments for each occurrence of any of the previously specified component types. Each instance assignment specifies the relationship between the component-instance parameters and the entity parameters. In addition, it specifies a port map detailing how the component-instance is connected to the internal signals or the external ports.

**Listing 3.** Architecture body.

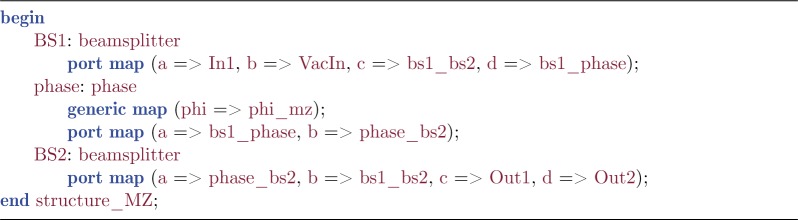

In the port map, each internal component port is assigned to either an entity port or a signal. Any instance *in* (*out*) port must be connected either to an entity *in* (*out*) port or to a signal that is connected to another instance's *out* (*in*) port.

**Listing 4.** Port map statement.

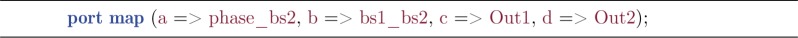

Each signal therefore connects exactly two ports: one instance input and one instance output *or* one instance input (output) and an entity input (output).

### (c) Parsing a network

Here, we present a simple algorithm to parse a general network into a circuit expression. We assume that the QHDL file has been preprocessed such that we have the lists of ports, components, instances, signals and port mappings in native data structures accessible to our algorithm.

1. We denote the list of internal signals by *S*. For each instance assignment *j*=1,2,…,*N* in the architecture body:
  — Generate the network triplet *Q*_*j*_=(**S**_*j*_,**L**_*j*_,*H*_*j*_) with the correct parametrization as specified in the generic map statement.
  — Generate the correctly ordered^2^ list of input port names *I*_*j*_ and the correctly ordered list of output port names *O*_*j*_ where each port name entry is of the form *instance-name:port-name*.
2. Concatenate all triplets 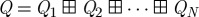 and similarly concatenate the input and output port lists *I*=*I*_1_+*I*_2_+⋯+*I*_*N*_ and *O*=*O*_1_+*O*_2_+⋯+*O*_*N*_.
3. For each internal signal *s*∈*S* concatenate the full circuit triplet *Q* with a single channel identity system 11_1_ resulting in 
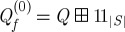
.
4. Now, each element in the full list of output ports *O* corresponds to an entry of the form *instance-name:port-name*. Make copies of *O*′=*O* and *S*′=*S* and iterate over all output ports in the following fashion:
  If the output port is connected to a global output (i.e. an entity output port), continue to the next entry.
  If the output port is connected to the *j*th signal in the *current* signal list *S*′, let *k* be the index of the output port in the *current* output port *O*′ list and update the model triplet 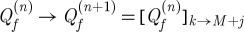, where *M*=|*O*′| is the length of the current output port list. Then, remove the *k*th entry from *O*′, and the *j*th entry of *S*′.
5. Now, let *M*_*f*_=|*O*′| and iterate over a copy of the input port list *I*′=*I* and a new copy of the signal list *S*′′=*S*:
  If the input port is connected to a global input (i.e. an entity input port), continue to the next entry.
  If the input port is connected to the *j*th entry of *S*′′ update 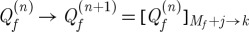, where *k* is the index of the current port in *I*′. Then, remove the *k*th entry of *I*′ and the *j*th entry of *S*′′.
6. By construction, the only remaining ports of our resulting triplet 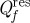 lead to global/entity ports. Iterating over *O*′ and the list of entity output ports *O*_E_, construct a suitable permutation *σ*_out_ that maps every output port index from *O*′ to the correct index of the entity output port. In a similar fashion, iterate over *I*′ and the list of entity input ports *I*_E_ to generate a permutation *σ*^−1^_in_, mapping the indices from *I*′ to the correct indices of the entity input ports within *I*_E_. Then, invert this permutation 
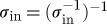
 to obtain a mapping from *I*_E_ to *I*′. Finally, the model triplet for the circuit is given by

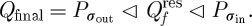

If one is interested in working with the actual network expressions as opposed to the more concrete level of the actual Hilbert space operators, there exist other, more complex, approaches to parsing a network, which directly yield simpler overall network expression. Combined with a sufficiently sophisticated set of circuit expression simplification rules, the above algorithm works just as well.

### (d) The quantum hardware description language workflow

The circuit design workflow relies heavily on symbolic computer algebra methods. Using symbolic algebra, rather than working with numerical matrix representations of all the operators appearing in the component parameter triplets, makes it possible to view the overall circuit (**S**,**L**,*H*) in an analytic form. It also allows the designer to defer choosing the values of numerical parameters, which could be convenient for optimization scenarios, as well as details such as the upper photon-number limits to use for truncated Fock spaces in numerical simulations.

In fact, we can define our own algebraic types, operations and simplification rules not just for Hilbert space operators and scalar coefficients, but also for circuit algebra components. This approach enables us to extend the hierarchical design principle even to our compiled QHDL component library, as will become clear in the following outline of the modelling workflow.

— *Circuit design*. In step 1, we visually compose the circuit using a schematic capture tool and then export to QHDL^3^ or directly describe the circuit in text-based QHDL. Because QHDL describes the connections between *functional* entities, it is not necessary at this stage to specify how referenced components are implemented.
— *Component model specification*. The QHDL file is then parsed to generate the circuit expression in which referenced components appear as symbols. This expression is stored in a library file along with information about model parameters and the component names of the referenced subcomponents. Note that a library file can be treated as a standalone entity for future circuit designs. When this file is imported at runtime, the referenced subcomponent models are dynamically loaded from their respective library files. Now, the full (**S**,**L**,*H*) parameter triplet can be generated by explicitly evaluating the circuit algebra operations. By means of the symbolic operator algebra, the final operator matrices and the Hamiltonian are still in fully symbolic form, which can be used to generate the quantum master equation or an appropriate stochastic differential equation in symbolic form. This allows for the application of analytical model reduction techniques before turning to purely numerical methods.
— *Numerical simulation*. Define all scalar model parameters and (truncated) Hilbert space dimensions, and compute the behaviour of the circuit.

In table 1, we list the necessary software tools to implement the QHDL circuit design workflow. We plan to publicly release our custom tools in the near future.

**Table 1.** List of software components necessary to realize our quantum hardware description language workflow.

requirement	our solution	alternatives
graphical *schematic capture* tool with VHDL/QHDL export capabilities	gschem and gnetlist from the gEDA suite [15]	graphical design tool from system modelling environments/modelling languages, such as Modelica [16]
*QHDL parser* that computes the circuit expression	a custom parser written in Python using the open source PLY [17] package	A parser for computing a circuit expression from the Modelica specification, written in, for example, Mathematica
*Symbolic computer algebra system* with support for: non-commutative operator algebra, commutative scalar coefficient algebra, operator-valued matrix algebra and the Gough–James circuit algebra	A custom computer algebra system written in Python [18] and interfacing with SymPy[19] for the scalar coefficient algebra	Mathematica[20] plus an implementation of the Gough–James circuit algebra
*Numerical backend* to convert symbolic operator expressions into matrices and simulate the system	custom algorithms for solving the master equation as well as quantum stochastic differential equations implemented in Python and C, using optimized numerical libraries for linear algebra [21,22]	The quantum optics toolbox [23] for Matlab [24] or similar library for modelling the dynamics of open quantum systems, such as QuTIP [25]

## 3. An example of the quantum hardware description language workflow

In this section, we present a detailed example applying the QHDL workflow to the design, analysis and simulation of an all-optical 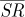-latch as recently proposed in the study of Mabuchi [7,8]. The elementary component models {(**S**_*j*_,**L**_*j*_,*H*_*j*_)} required for this circuit are the following.

— *Beamsplitters*

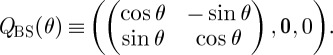
— *Phase-delays* *U*(*ϕ*)≡(*e*^*iϕ*^,0,0).
— *Coherent displacements* *W*(*α*)≡(1,*α*,0), which models a laser source outputting a coherent field with amplitude 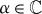.
— *Kerr-nonlinear cavity* (here, a unidirectional ring cavity with two input/output ports)

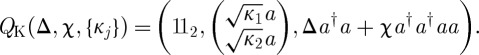

As the circuits we discuss here are meant to be used as logical gates in larger circuits, we need not include the laser sources in our circuit schematics. Instead, they can easily be added at the level of the circuit algebra by feeding a concatenated block of laser displacements (sources) into the full network 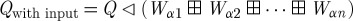.

### (a) The two-cavity pseudo-NAND latch

**Figure 3.**
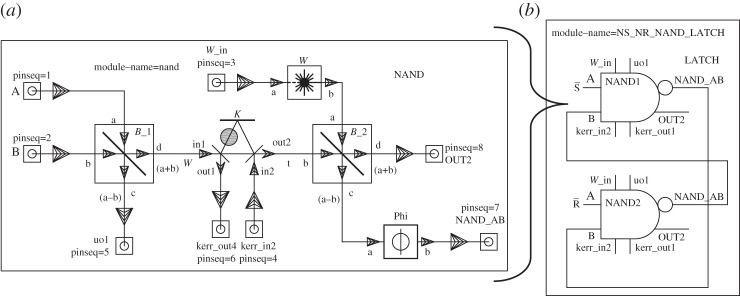
Pseudo-NAND circuit schematic (*a*) as created with gschem and its device symbol embedded as a component in an SR-NAND-latch circuit (*b*).

**Figure 4.**
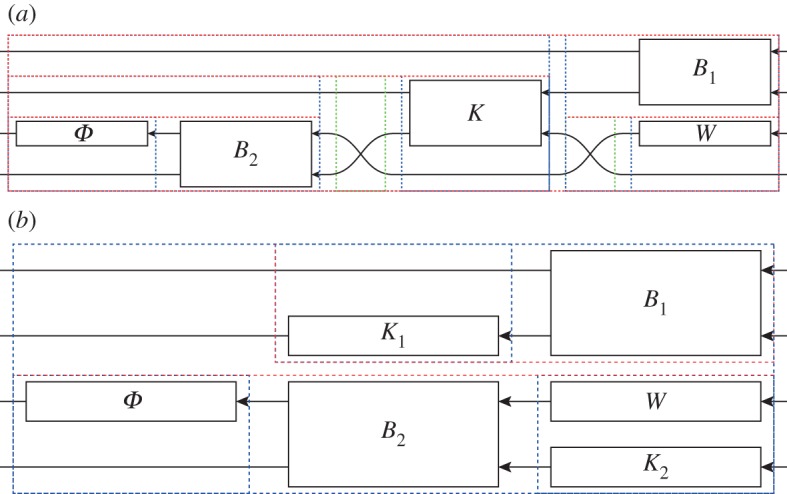
Pseudo-NAND circuit expression visualizations. As can easily be verified visually, the simplified expression follows from decomposing 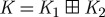 and ‘pulling’ *K*_2_ down into the fourth row. These expression simplifications are automatically performed by our symbolic circuit algebra software. (*a*) Circuit expression (3.1) as generated by the QHDL parser; (*b*) simplified expression (3.2).

We have recently proposed [8] several different optical circuits to realize three classical logic gates: an AND gate, a NOT gate with integrated fanout of two and a combined (but imperfect) NAND gate (figure 3*a*), which in the following we will call a pseudo-NAND gate, as it works properly only when, at any given time, at least one input is in the ‘on’ state. The first two of these gates used in sequence also realizes a NAND gate, but the advantage of the pseudo-NAND is that it requires only a single Kerr-nonlinear cavity component. The QHDL workflow can be readily applied to design the pseudo-NAND circuit and automatically generate the circuit expression in terms of its components^4^
3.1
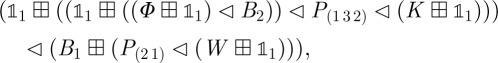
where the beamsplitter symbols are defined by *B*_1_≡*Q*_BS_(*π*/4), *B*_2_≡*Q*_BS_(*θ*), the output correction phase is *Φ*≡*U*(*ϕ*), the constant coherent displacement component is *W*≡*W*(*β*) and the Kerr cavity is given by *K*≡*Q*_*K*_(Δ,*χ*,*κ*_1_=*κ*_2_=*κ*). The network expression (3.1) looks complicated but can be verified easily by comparing its visual representation (figure 4*a*)^5^ with the original circuit schematic. Moreover, since the scattering matrix of *K* is in block-diagonal form, it is possible to decompose the cavity component 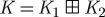, where the Hamiltonian of *K* can be assigned to either of the two blocks. Upon substituting this decomposable form into the expression, the automatic expression simplification built into our circuit algebra implementation yields the following form:
3.2
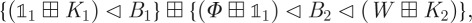
which is visually represented in figure 4*b*.

The numerical model parameters as given in Mabuchi [8] are *θ*=0.891, *χ*=−5/6, Δ=50, *κ*=25, *ϕ*=2.546 and the auxiliary constant input amplitude is given by *β*=−34.289−11.909i. The coherent input amplitudes corresponding to the logical signals ‘on’ and ‘off’ are then given by *α*=22.6274 and 0, respectively.

As in classical circuit theory, two NAND gates in a mutual feedback configuration, as shown in figure 3*b*, can be used to realize a latch with inverted inputs 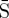 and 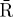. A latch features controllable bistable behaviour and thus realizes a single-bit memory unit. It has two inputs: S(ET) and R(ESET), which can be activated individually to control the internal logical state to ‘on’ or ‘off’, respectively. Ideally, when both S and R are ‘off’ (HOLD-condition), the internal state remains stable. In practice, quantum fluctuations and noisy inputs lead to spontaneous switching between the two internal states. One of the design goals is thus to decrease the rate at which this spontaneous switching occurs. The QHDL code as produced by gnetlist [15] (slightly edited to be more concise) can be found in listing 5, and the circuit component library file generated by the QHDL parser is presented in listing 6 in appendix B. Substituting the individual component models into the circuit expression yields the full triplet (**S**_0_,**L**_0_,*H*_0_) for the latch. Finally, after feeding in the coherent input signals 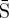 and 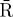 into their respective ports, 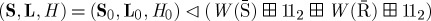, the parameters assume the following form:
3.3
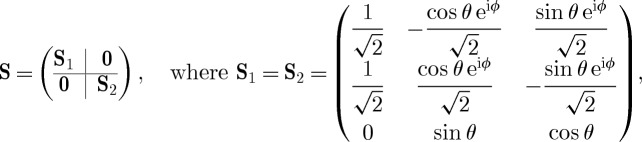

3.4
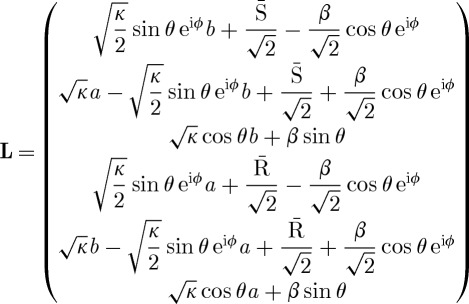

and
3.5
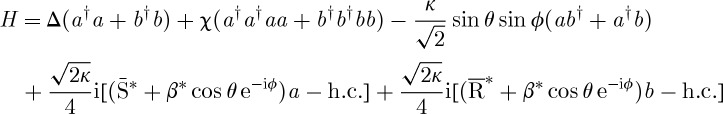
Owing to the symmetry of the underlying circuit model, the model parameters are invariant under exchange of the two pseudo-NAND gates, which corresponds to simultaneously exchanging 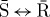, 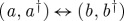, 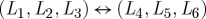 and 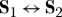. This symmetry suggests that the most likely candidates for the internal logical states ‘on’ and ‘off’ correspond to the case where one internal cavity mode is in a high power state and the other one in a low power state and the opposite case, obtained by exchanging the cavity states. This is indeed the case, and in fact it follows from the basic way in which we have designed our pseudo-NAND gate; ‘on’ ⇔{NAND1 cavity power is low, NAND2 cavity power is high} and ‘off’ ⇔ (NAND1 cavity power is high, NAND2 cavity power is low).

**Listing 5.** Quantum hardware description language source for the pseudo-NAND latch.

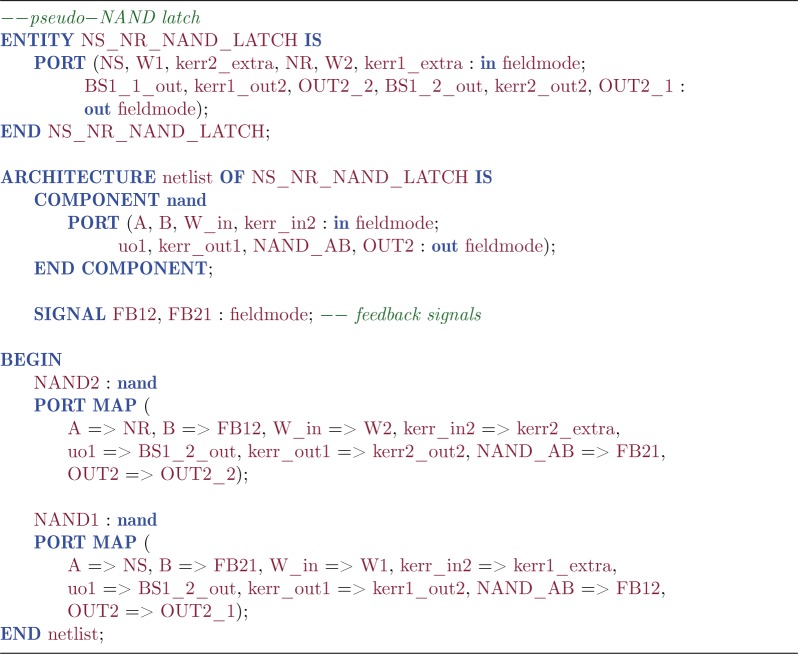

**Figure 5.**
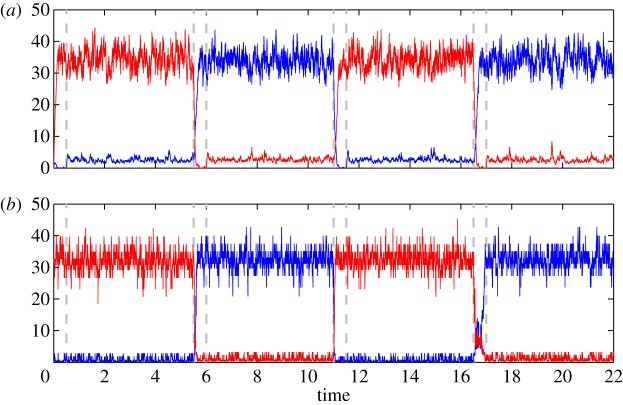
Simulated input sequence for the full pseudo-NAND latch model (*a*) and our reduced model (*b*). The red trace (lighter, in greyscale) is given by 〈*a*^†^*a*〉 and the blue trace presents 〈*b*^†^*b*〉. The SET and RESET input conditions, marked by the short intervals between dashed vertical lines, induce transitions to their respective target latch states: ‘on’ corresponds to the *a*-mode being in a high photon number state, while ‘off’ corresponds to a high photon number in mode *b*.

To understand our model's dynamic behaviour, we turn to numerical methods. The simulation of this model is carried out by representing the operators as numerical matrices in a truncated product basis of Fock states of total dimension *N*^2^=75^2^=5625.^6^ We carried out a large number of quantum jump trajectory simulations [23,26] with the following sequence of alternating input conditions: 0.5 time units of SET, 5 units of HOLD, 0.5 units of RESET, 5 units of HOLD (repeated twice). Figure 5*a* presents a typical simulated trace where the system is subjected to this sequence of input conditions. We generally find that the SET and RESET input conditions successfully drive the system into the desired cavity states, while the cavities remain in their states during the HOLD condition. Although a simulation of the full master equation is feasible using current HPC hardware and sparse matrix storage [8], quantum jump simulations exhibit the inherently bistable nature of our synthesized latch more clearly.

### (b) Model reduction in the (***S***,***L***,*H*) context

As the latch could readily be used as a component in more complex circuits, such as flip-flops or even quantum memories [5,6], it would be highly desirable to reduce the Hilbert space dimension *N*^2^ required to represent it. Because we are working with quantum circuit models, we are ultimately limited by exponential scaling of the state space with the number of components (although it may be possible to develop efficient simulation procedures when components are only weakly entangled, as should be the case in ultra-low-power classical signal processing). However, there is clearly much to be gained by developing accurate model reduction procedures that allow us to replace high-dimensional *ab initio* models for components within a circuit by much lower-dimensional effective models. Such model-reduction strategies could presumably be applied hierarchically. As in the classical theory of signals-and-systems, there are many potential strategies for dimensional reduction of quantum input–output models. For the case of components or subcircuits whose input–output behaviour admits a simplified description as certain parameter ratios become large, a recently derived limit theorem [27,28] can be used, as demonstrated in Kerckhoff *et al*. [5,6]. Here we describe an empirical approach similar to the classical strategy of approximating Markov chains [29], which uses numerical simulation and statistical analysis to derive a reduced (**S**,**L**,*H*) model for the pseudo-NAND latch (for which no simplifying parameter limits are known).

Our approach is based on the assumption that the device state can be inferred with reasonable accuracy from a small number of observables. We can then construct a dynamic model just in terms of these parameters [30]. By generating many quantum jump trajectories for the full (**S**,**L**,*H*) model, we generated many time series for the expectation values of the cavity field photon numbers 〈*a*^†^*a*〉 and 〈*b*^†^*b*〉 under the three valid input conditions {HOLD,SET,RESET}. We can now coarse-grain the two-dimensional space of expectation values and associate an internal model state *i*∈{1,2,…,*M*} with each bin that is actually visited during the trajectory simulations, but owing to the high correlations between the cavity photon numbers, we are actually able to obtain good results by performing this coarse-graining or ‘binning’ procedure in terms of the single quantity

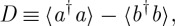
implying that within the two-dimensional configuration space, our system always stays fairly close to a one-dimensional submanifold.

By analysing the observed transitions between these reduced states for each input condition *ξ*∈{HOLD,SET,RESET}, we calculate an empirical estimate 
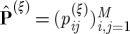
 of the conditional transition probabilities 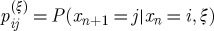 and thus model the system in terms of a discrete time Markov chain with a set of conditional transition probabilities for each particular input condition *ξ*. The time step *δt* of the discrete Markov chain corresponds to the interval at which we sampled our original continuous-time system. We now wish to get back to a description that is compatible with our (**S**,**L**,*H*) formalism. In the following, we briefly outline a procedure to do this: for a temporally homogeneous Markov jump process with an even number of states *i*∈{1,2,…,*M*} and transition rate matrix^7^ 
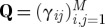
, we can define a *K*-channel (**S**_0_,**L**_0_,*H*_0_) model with states corresponding directly to the Markov process states {|1〉,|2〉,…,|*M*〉} via
3.6
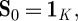

3.7
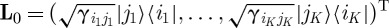

and
3.8
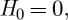
where the components of **L**_0_ drive transitions {*i*_*k*_→*j*_*k*_,*k*=1,2,…,*K*} and *K* is given by the number of positive transition rates *γ*_*ij*_>0. By construction, as one may verify by writing down the master equation, this system always collapses into a purely classical mixture of the coarse-grained states. Equivalently, in a quantum jump trajectory simulation, after the first quantum jump, the state is always given by a single such state. In fact, in such a trajectory simulation, this system behaves exactly like the original Markov jump process. Neglecting for now that our original model has three different input conditions *ξ*∈{HOLD,SET,RESET} and thus three different conditional transition matrices 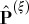, we first discuss how to move from the discrete time Markov chain model to a continuous time Markov jump process. Rephrasing this question, we can ask the following: is there a Markov jump process with conditional transition probability matrix **P**(*t*) that ‘looks like’ our Markov chain when stroboscopically probed at fixed time intervals *δt*? If our Markov chain has transition matrix 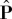, then we need to determine a generator matrix **Q** such that
3.9
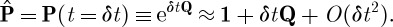
If our sample interval *δt* is sufficiently small, we may define
3.10
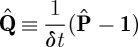
as an approximation to the conditional rate matrix. We now carry out the procedure outlined earlier to create a model (**S**_0_,**L**_0_,*H*_0_) that realizes the HOLD condition. The transition rates of the HOLD condition alone lead to a system that has two bistable clusters of states with low-state indices and high-state indices, respectively.

To account for the input-controlled switching in the SET and RESET conditions, we extend our model by concatenating it with a second model that explicitly includes the input fields 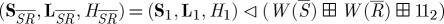. Hence, in the SET and RESET conditions, the HOLD transitions continue, but we drive further transitions through this additional component. Here, (**S**_1_,**L**_1_,*H*_1_) is given by
3.11
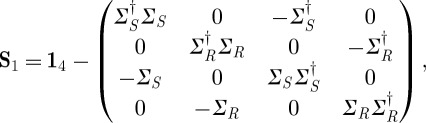

3.12
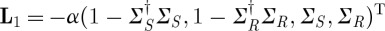

and
3.13
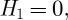
where the ‘drift’ operators *Σ*_*S*_ and *Σ*_*R*_ are defined by

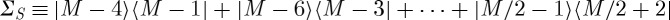
and

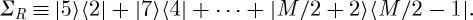
For our simulation, we chose *M*=38≪*N*^2^, but the general ansatz works for a range of different *M*=4*k*+2 with sufficiently large *k*. The drift operators satisfy 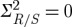, as well as the projection relations 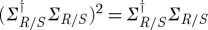, 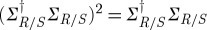 and 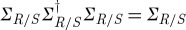. These relations suffice to show that **S**_1_, as defined earlier, is indeed unitary. To make sense of the effect of this extension to our model, consider now what happens for the different input conditions. In the HOLD condition 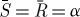, the input fields cancel out all elements of the coupling vector **L**_1_ and we have 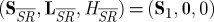, i.e. the transition dynamics of our full system 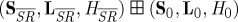 are simply given by those of (**S**_0_,**L**_0_,*H*_0_) alone.

**Figure 6.**
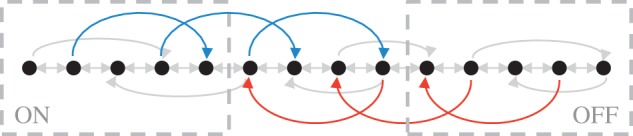
Schematic visualization of the state space and the transitions of the reduced model. The SET transitions (red, from right to left) introduce a drift that drives the system to the states on the left, corresponding to the logical ‘ON’ state of the latch. The RESET transitions (blue, from left to right) have the opposite effect. The HOLD transitions (depicted in grey) are always active, but, in the absence of additional SET and RESET transitions, only very rarely lead to a switch of the logical latch state.

In the SET condition, however, we have 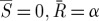 and thus

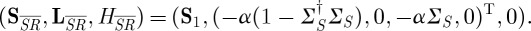
The full system 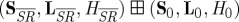 now features the drift operator as an additional transition operator^8^ −*αΣ*_*S*_ which induces transitions {*M*−1→*M*−4,*M*−3→*M*−6,…,*M*/2+2→*M*/2−1} with constant rate |*α*|^2^. Together with the HOLD transitions, these lead to a drift from states with high index (corresponding to the logical ‘off’ state of the latch) to those with low index (‘on’). On the other hand, in the RESET condition, the situation is reversed. Now the other transition operator of **L**_1_ is cancelled out and the non-zero transition operator −*αΣ*_*R*_ drives transitions in the reverse direction {2→5,4→7,…,*M*/2−1→*M*/2+2}, again with constant rate |*α*|^2^. In figure 6, we visualize the transition structure of the model schematically.

Note also that we can emulate the state-dependent coherent output field(s) of the latch by concatenating a triplet (**S**_out_,**L**_out_,*H*_out_) that re-routes bias input fields via state-dependent scattering into one or more output channels. For example, we could use 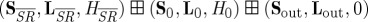, where
3.14
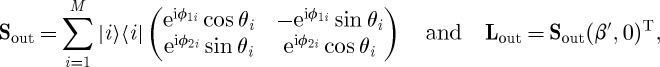
where *β*′ is the complex amplitude of a bias field and the parameters {*θ*_*i*_,*ϕ*_1*i*_,*ϕ*_2*i*_} are chosen such that the outputs of (**S**_out_,**L**_out_,0) vary as desired with the internal state |*i*〉. Having thus created a reduced model that mimics the desired input–output behaviour in (**S**,**L**,*H*) form, we can use it to replace the full latch model in more complex circuits. If we had already specified a QHDL file for such a circuit, we could simply replace^9^ the referenced latch component with the reduced model component. Re-parsing this modified QHDL file would then yield a computationally more tractable model for simulations.

## 4. Conclusion

In this paper, we have described the use of QHDL to facilitate the analysis, design and simulation of complex networks constructed from interconnected quantum optical components. We have also presented a parsing algorithm for obtaining quantum equations of motion from the QHDL description. QHDL can be used as the basis for a schematic capture workflow for designing quantum circuits that automates many of the conceptually challenging and computationally demanding aspects of quantum network synthesis. As QHDL inherits the hierarchical structure of VHDL, its use may facilitate the crucial development of hierarchical model reduction methods for quantum nonlinear photonics.

Important future directions for QHDL research include simulation strategies for exploiting weak entanglement among components, stability analysis and design optimization of QHDL-based models [31], and the incorporation of techniques from static program analysis and formal verification to assist in the design of complex, hierarchically defined photonic components. While we have emphasized classical photonic logic [8] as a tutorial paradigm for QHDL in this paper, emerging ideas in quantum information processing and quantum sensing/metrology may provide even more compelling applications for QHDL as a convenient and extensible modelling framework.

## Footnotes

1 It is important to note that the Gough–James circuit algebra cannot be used to build dynamical systems from static components; e.g. it cannot create the Fock space and operator algebra for an optical resonator mode as an automatic result of cascading beamsplitters and phase-shifts in the configuration of a ring cavity. All such dynamic components therefore must be implemented as primitive (**S**,**L**,*H*) models.

2 As defined via the component declaration in the architecture head.

3 In our case, we have modified the VHDL exporting functionality of the gEDA toolsuite to generate QHDL.

4 Here, we represent a permutation  just by its image tuple (*σ*(1)*σ*(2)⋯*σ*(*n*)).

5 These visualizations were automatically generated using another software tool we have implemented.

6 That is, each individual cavity basis is given by {|0〉,|1〉,…,|*N*−1〉}.

7 **Q** is also often referred to as the *generator matrix*.

8 The second non-zero element of , which is a projection operator, does not affect the transition dynamics, because our system is never in a superposition of two states.

9 In principle, it should be possible to include the reduced model as an alternative architecture for the latch entity and to select whether or not to use it in place of the full model at compile-time using a VHDL configuration file. However, this would require some enhancements to the QHDL parser to correctly handle the *K* extra (vacuum) input ports required by the reduced model to drive spontaneous transitions among the internal states.

## Appendix A. Reduced parameters in the case of signal feedback

Upon feeding the *k*th output channel of a system *Q*=(**S**,**L**,*H*) back into its *l*th input, we get a system  with one less channel cdim[*Q*]_*k*→*l*_=cdim *Q*−1, where effective parameters are then given by [1]

A1

and

A2

Here we have written  as a shorthand notation for the matrix **S** with the *k*th row and *l*th column removed and similarly  is the vector **L** with its *k*th entry removed. These resulting parameters fulfil the conditions^10^ for circuit components. Moreover, they have shown that in the case of multiple feedback loops, the result is independent of the order in which the feedback operation is applied.^11^

## Appendix B. Latch circuit library file

**Listing 6.** Python [18] source for the pseudo-NAND latch circuit library component.
